# 4179 Use of tobacco products and their association with wheezing among adult current tobacco users in the US – Findings from The Population Assessment of Tobacco and Health Study (2015-2016)

**DOI:** 10.1017/cts.2020.188

**Published:** 2020-07-29

**Authors:** Liane M Schneller, Zahíra Quiñones-Tavárez, Maciej Goniewicz, Zidian Xie, Scott McIntosh, Richard O’Connor, Deborah J. Ossip, Irfan Rahman, Dongmei Li

**Affiliations:** 1 University of Rochester Medical Center

## Abstract

OBJECTIVES/GOALS: Wheezing has been shown to be associated with use of cigarettes, and more recently, electronic nicotine delivery systems (ENDS). This study assessed the association of poly use of tobacco products with wheezing among a national representative sample of US adult current tobacco users. METHODS/STUDY POPULATION: Data from the Population Assessment of Tobacco and Health (PATH) Study Wave 3 (W3) were used. Weighted prevalences of self-reported wheezing and related respiratory symptoms for non-users compared to users of cigarettes, ENDS, cigars, and any combination of these products (poly use of tobacco products) were presented for 28,082 adults. The cross-sectional association of tobacco use with self-reported wheezing and other related respiratory symptoms was assessed using weighted multivariable and ordinal logistic regression with consideration of complex sampling design. RESULTS/ANTICIPATED RESULTS: Most adults who reported on wheezing symptoms did not currently use cigarettes, ENDS or cigars (79%), 15% used cigarettes, 3% used a combination of cigarettes, ENDS and cigars, 1% used ENDS, and 1% used cigars. Significantly higher odds of ever had wheezing or whistling in chest at any time in the past was observed among current cigarette (adjusted OR: 2.62, 95%CI: 2.35, 2.91), ENDS (1.49, 95%CI: 1.14, 1.95), and poly users (2.67, 95%CI: 2.26, 3.16) compared to non-users. No differences were seen for cigar use. Polytobacco use was associated with a higher odds of ever wheezing when compared to ENDS (1.61, 95%CI: 1.19, 2.17) and cigar use (2.87, 95%CI: 1.93, 4.26), but not cigarettes. DISCUSSION/SIGNIFICANCE OF IMPACT: Wheezing is associated with the use of cigarettes, ENDS, or any combination of cigarette, ENDS and cigars likely due to the inhalation of noxious chemicals and gases found in the smoke of cigarettes and ENDS that are likely to increase the odds of experiencing wheezing. CONFLICT OF INTEREST DESCRIPTION: MLG serves as a paid consultant for Johnson & Johnson and has received research grant from Pfizer, manufacturers of smoking cessation medications. The other authors have no conflicts to declare.

